# Crossed Cerebellar Diaschisis in Acute Isolated Thalamic Infarction Detected by Dynamic Susceptibility Contrast Perfusion MRI

**DOI:** 10.1371/journal.pone.0088044

**Published:** 2014-02-05

**Authors:** Alex Förster, Hans U. Kerl, Johannes Goerlitz, Holger Wenz, Christoph Groden

**Affiliations:** Department of Neuroradiology, Universitätsmedizin Mannheim, University of Heidelberg, Mannheim, Germany; University Hospital La Paz, Spain

## Abstract

**Purpose:**

Crossed cerebellar diaschisis (CCD) is a state of neural depression caused by loss of connections to injured neural structures remote from the cerebellum usually evaluated by positron emission tomography. Recently it has been shown that dynamic susceptibility contrast perfusion weighted MRI (PWI) may also be feasible to detect the phenomenon. In this study we aimed to assess the frequency of CCD on PWI in patients with acute thalamic infarction.

**Methods:**

From a MRI report database we identified patients with acute isolated thalamic infarction. Contralateral cerebellar hypoperfusion was identified by inspection of time to peak (TTP) maps and evaluated quantitatively on TTP, mean transit time (MTT), cerebral blood flow and volume (CBF, CBV) maps. A competing cerebellar pathology or an underlying vascular pathology were excluded.

**Results:**

A total of 39 patients was included. Common symptoms were hemiparesis (53.8%), hemihypaesthesia (38.5%), dysarthria (30.8%), aphasia (17.9%), and ataxia (15.4%). In 9 patients (23.1%) PWI showed hypoperfusion in the contralateral cerebellar hemisphere. All of these had lesions in the territory of the tuberothalamic, paramedian, or inferolateral arteries. Dysarthria was observed more frequently in patients with CCD (6/9 vs. 6/30; OR 8.00; 95%CI 1.54–41.64, p = 0.01). In patients with CCD, the median ischemic lesion volume on DWI (0.91 cm^3^, IQR 0.49–1.54 cm^3^) was larger compared to patients with unremarkable PWI (0.51 cm^3^, IQR 0.32–0.74, p = 0.05). The most pronounced changes were found in CBF (0.94±0.11) and MTT (1.06±0.13) signal ratios, followed by TTP (1.05±0.02).

**Conclusions:**

Multimodal MRI demonstrates CCD in about 20% of acute isolated thalamic infarction patients. Lesion size seems to be a relevant factor in its pathophysiology.

## Introduction

At the beginning of the 20th century the term “diaschisis” was introduced by the russian neurologist and neuropathologist Constantin von Monakow to describe a state of neural depression in the brain caused by loss of connections to injured neural structures remote from the affected brain area [1]. The pathophysiological concept of diaschisis is nowadays widely accepted and many articles concerning different aspects of this phenomenon have been published meanwhile [2], [3]. In most of these studies positron emission tomography (PET) has been used to demonstrate a hypometabolism or hypoperfusion in brain areas distant from the actual lesion like the ipsilateral cerebral cortex [4], [5] or the contralateral cerebellum [6]–[10]. The latter phenomenon has also been referred to as crossed cerebellar diaschisis (CCD). More recently, some authors demonstrated that MR perfusion techniques may also be feasible to detect CCD in supratentorial intracerebral hemorrhage and ischemic infarction [11]–[13].

In particular in the thalamus with its numerous connections to different sensory pathways, the motor system including basal ganglia and brainstem, as well as the limbic system, lesions may give rise to signs and symptoms like aphasia, hemineglect, or ataxia among others which are thought to represent a cortical dysfunction by impaired thalamocortical connections [4]. Acute thalamic infarctions are usually unilateral and account for about 11–14% of acute ischemic stroke in the posterior circulation [14], [15]. The arterial blood supply of the thalamus can be subdivided into four major vascular territories: (1) the tuberothalamic artery (syn. polar artery) emanating from the posterior communicating artery (PComA), (2) the paramedian arteries (syn. thalamoperforating arteries) arising from the P1 segment of the posterior cerebral artery (PCA) as well as (3) the inferolateral arteries (syn. thalamogeniculate arteries) and (4) the lateral and medial branches of the posterior choroidal artery, from the P2 segment of the PCA [16]. Infarctions in these distinct vascular territories can be found in varying frequency and are associated with typical, well-defined clinical syndromes [16], [17].

Until today there are no studies systematically evaluating the presence and extent of CCD in acute isolated thalamic infarctions. We therefore aimed to assess the frequency of CCD on perfusion-weighted MRI (PWI) and the degree of hemodynamic alterations in a retrospective series of patients with acute isolated thalamic infarction collected over 9 years who were evaluated by a standard stroke MRI protocol including diffusion-weighted MRI (DWI) and dynamic susceptibility contrast PWI. Furthermore, we sought to compare the clinical presentation in patients with and without CCD.

## Materials and Methods

### Patients

This study was approved by the local institutional review board (Medizinische Ethikkommission II der Medizinischen Fakultät Mannheim) and did not require consent. Consent was not required by our IRB for this de-identified database (Perfusion-weighted imaging in Isolated Thalamic Infarction - PITI) due to the retrospective nature of the study and the lack of patient interaction. From a prospectively maintained MRI report database (Syngo Data Manager – SDM), we identified 698 patients with suspected acute ischemic stroke who underwent a standard stroke MRI protocol including PWI (2005–2013). Among these, 46 (6.6%) had an acute isolated ischemic infarction in the thalamus. Of these, 2 patients were excluded due to cerebellar hypoperfusion restricted to the territory of the posterior inferior cerebellar artery, 3 patients due to occlusion of the posterior cerebral artery and marked hypoperfusion, and finally, 2 patients because of suboptimal MRI quality due to motion artifacts. The remaining 39 (5.6%) patients formed the study population and were retrospectively studied with regard to demographic details and clinical symptoms as abstracted from the case records as well as MRI findings. Standardized clinical work-up included assessment of cardiovascular risk factors, extra- and transcranial Doppler−/duplex sonography, 24-hour electrocardiogram monitoring and transthoracic or transesophageal echocardiography as well as laboratory tests according to stroke unit standard requirements in Germany [18].

### Magnetic Resonance Imaging

Magnetic resonance imaging was performed on a 1.5-T MR system (Magnetom Avanto or Sonata, Siemens Medical Systems, Erlangen, Germany) at admission. A standardized protocol was used in all patients including (1) transverse, coronal and sagittal localizing sequences followed by transverse oblique contiguous images aligned with the inferior borders of the corpus callosum (applied on sequences 2–5 and 7); (2) T1-weighted spin echo sequence; (3) T2-weighted turbo spin echo sequence; (4) isotropic diffusion-weighted echo-planar spin-echo sequence (DWI); (5) fluid attenuated inversion recovery (FLAIR) sequence; (6) a time-of-flight MRA; and (7) PWI following the first pass of contrast bolus through the brain. Perfusion-weighted imaging was acquired using a gradient-echo echo planar imaging sequence. The contrast agent gadoteric acid (Dotarem, Guerbet, Aulnay-sous-Bois, France) was bolus injected by a power injector (Spectris MR injection system, Medrad, Volkach, Germany) with a dose of 0.1 mmol/kg of body weight at a rate of 4 ml/sec.

### Postprocessing

The postprocessing of the perfusion-weighted raw images was performed by a specific software, Signal Processing In NMR (SPIN, The MRI Institute for Biomedial Research, Detroit, USA) [19]. Deconvolution with singular value decomposition (SVD) was used to create quantitative maps of mean transit time (MTT), cerebral blood flow (CBF), and cerebral blood volume (CBV). The position of the arterial input function (AIF) was automatically determined by using the maximum concentration (Cmax), time to peak (TTP) and first moment MTT (fMTT). The concentration-time curve for arteries has short fMTT, short TTP and high Cmax. Twenty voxels, which best fit these properties were selected. Then the concentration-time curves of these voxels were averaged, smoothed and truncated to avoid the second pass of the tracer.

### Image Analysis

Acute ischemic lesion size was measured on DWI by manually delineated region of interest (ROI), summation of these areas in cm^2^ on each section and multiplication by the interslice spacing, to determine the volume in cm^3^ by use of OsiriX, a multidimensional image navigation and display software [20].

To obtain information about hemodynamic alterations, calculated time to peak (TTP) images demonstrating the delay of the contrast agent arrival in the brain parenchyma were used for visual analysis. Cases with cerebellar hypoperfusion contralateral to the thalamic infarction not confined to a specific vascular territory were identified by visual inspection of the TTP maps. For the diagnosis of crossed cerebellar diaschisis a decrease in perfusion on PWI on the basis of increased TTP of the contralateral cerebellar hemisphere was mandatory. In addition, the generated perfusion maps were quantitatively assessed by use of SPIN: a ROI encompassing the complete hypoperfused cerebellar hemisphere was placed on the generated maps (CBF, CBV) as well as mirrored to the contralateral unaffected hemisphere. Finally, ratios between the physiological estimates of the affected hemisphere and the contralateral mirror ROI were then determined.

A competing cerebellar pathology or a possible underlying abnormality of the vertebrobasilar system was excluded on DWI, T1- and T2-weighted images, and MR angiography respectively.

### Statistical Analysis

All statistical analyses were performed using Statistical Product and Service Solutions (SPSS) statistics for Windows (Release 17.0; SPSS, Chicago, IL, USA). Descriptive data was analyzed using either the Mann-Whitney *U* test or chi^2^ based tests as appropriate. Comparison of lesion size on DWI in patients with and without CCD was performed using the Mann-Whitney *U* test. Association of CCD and presence of ischemic lesions on DWI in the different vascular territories of the thalamus was determined by use of chi^2^ based tests. Correlations between ischemic lesion size on DWI and mean signal ratios of TTP, MTT, CBF, and CBV were determined by the Spearman correlation coefficient. All statistics was performed with a 0.05 level of significance.

## Results

### Baseline Characteristics and Clinical Presentation

In the final analysis 39 of 698 (5.6%) patients were included. The median age was 72 years (IQR 63–79 years); 20 (51.3%) patients were male, and 19 (48.7%) patients were female. Most commonly observed clinical symptoms were hemiparesis (53.8%), hemihypaesthesia (38.5%), dysarthria (30.8%), aphasia (17.9%), ataxia (15.4%), oculomotor dysfunction (12.8%), and altered vigilance (10.3%) whereas other symptoms like disorientation, apraxia or vertigo were observed only occasionally. For details see Table 1.

**Table 1:** Demographic characteristics, ischemic lesion size and localization on diffusion-weighted images (DWI) as well as frequency of clinical symptoms in isolated thalamic infarction patients with crossed cerebellar diaschisis (CCD) and normal PWI.

	All, n = 39	CCD, n = 9	Normal PWI, n = 30	OR; 95%CI	p
**Age, years (median, IQR)**	72 (63–79)	75 (67.5–81.5)	72 (57–76.25)		0.13
**Male sex (n, %)**	20 (41.3)	3 (33.3)	17 (56.7)	0.38; 0.08–1.83	0.22
**Time between onset and MRI,**	325 (241–480)	343 (106–420)	321 (244.75–480)		0.54
**minutes (median, IQR)**					
**DWI lesion size, cm^3^ (median, IQR)**	0.65 (0.35–0.91)	0.91 (0.49–1.54)	0.51 (0.32–0.74)		0.05
**DWI lesion localization (n, %)**					
** Tuberothalamic artery territory**	5 (12.8)	3 (33.3)	2 (3.3)		0.16
** Paramedian artery territory**	17 (43.6)	4 (44.4)	13 (43.3)		
** Inferolateral artery territory**	16 (41.0)	2 (22.2)	14 (46.7)		
** Posterior choroidal artery territory**	1 (2.3)	0	1 (3.3)		
**Clinical symptoms (n, %)**					
** Dysarthria**	12 (30.8)	6 (66.7)	6 (20.0)	8.00; 1.54–41.64	**0.01**
** Hemiparesis**	21 (53.8)	4 (44.4)	17 (56.7)	0.61; 0.14–2.74	0.71
** Hemihypaesthesia**	15 (38.5)	2 (22.2)	13 (43.3)	0.37; 0.07–2.11	0.44
** Hemiataxia**	6 (15.4)	2 (22.2)	4 (13.3)	1.86; 0.28–12.31	0.61

### MRI Analysis

All patients underwent MRI within a median time of 325 minutes (IQR 241–480 minutes) after symptom onset. Diffusion weighted imaging demonstrated an acute ischemic infarction in the thalamus in the territory of the tuberothalamic artery in 5 (12.8%), the paramedian artery in 17 (43.6%), the inferolateral arteries in 16 (41.0%) patients and the posterior choroidal arteries in 1 (2.6%) patient. The right side was affected in 15 (38.5%), and the left side in 24 (61.5%) patients. The ischemic lesions had a median volume 0.65 cm^3^ (IQR 0.35–0.91 cm^3^).

**Figure 1:**
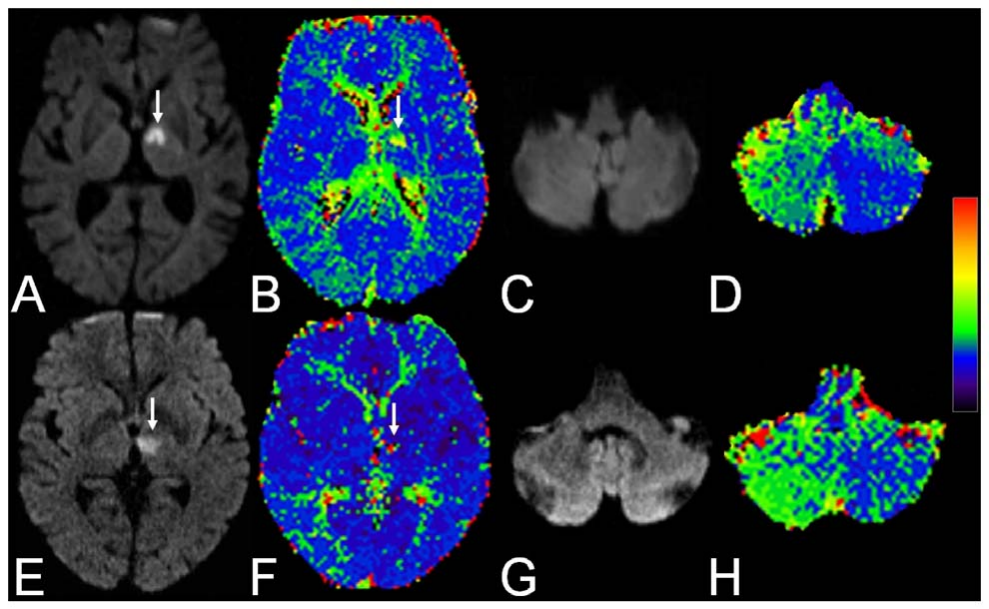
Two examples of crossed cerebellar diaschisis. 1. A 75-year-old female patient with reduced vigilance, dysarthria, and right-sided hemihypaesthesia: *A* Trace diffusion-weighted image shows an acute ischemic lesion (arrow) in the territory of the left tuberothalamic artery with *B* matching hypoperfusion (arrow) on the time to peak (TTP) map. *C* In the cerebellum trace DWI is unremarkable while *D* TTP demonstrates hypoperfusion in the contralteral cerebellar hemisphere. 2. A 65-year-old female patient with right-sided hemiparesis: *E* Trace DWI shows an acute ischemic lesion (arrow) in the territory of the left paramedian artery with *F* minor hypoperfusion (arrow) on TTP. *G* In the cerebellum trace DWI is unremarkable while *H* TTP demonstrates hypoperfusion in the contralateral cerebellar hemisphere.

In 9 patients (23.1%) PWI showed a hypoperfusion in the contralateral cerebellar hemisphere not confined to a specific vascular territory. For examples see Figure 1. In none of these patients a competing cerebellar pathology or an underlying abnormality of the vertebrobasilar system was observed. Quantitatively, the most pronounced changes were found in MTT (1.06±0.13) and CBF signal ratios (0.94±0.11), followed by TTP (1.05±0.02), while CBV (1.00±0.10) revealed no substantial changes.

Lesion size on DWI did not correlate with signal ratios of TTP (r_S_ = 0.30, p = 0.43), MTT (r_S_ = 0.22, p = 0.58), CBF (r_S_ = −0.22, p = 0.58), nor CBV (r_S_ = 0.25, p = 0.52).

### Comparison of Patients with and without Crossed Cerebellar Diaschisis

With regard to the clinical presentation no significant differences were observed except for a higher rate of dysarthria in patients with CCD (p = 0.01). Patients with crossed cerebellar diaschisis showed a trend towards a larger median volume of the acute ischemic lesion on DWI in comparison to patients with unremarkable PWI (p = 0.05). For details see Table 1.

## Discussion

Crossed cerebellar diaschisis is a well-defined phenomenon probably caused by interruption of cortico-ponto-cerebellar fibers [2], [21], [22] or the dentatorubrothalamic pathway [8] and has been demonstrated in a large variety of brain diseases like ischemic infarction [7], [23], intracerebral hemorrhage [9], [24], [25], and brain tumors [26]. Positron emission tomography is the gold standard for detection of CCD although it has been shown that dynamic susceptibility contrast perfusion MRI also may be feasible to demonstrate the hemodynamic alterations in CCD [11], [12].

In the present study, we were able to demonstrate CCD in about 20% of patients with isolated thalamic infarctions. This frequency is in line with a previously published study on detection of CCD with perfusion MRI in supratentorial infarction reporting a rate of 15.61% [12]. In contrast to this, the frequency is much lower in comparison to another study evaluating perfusion MRI in CCD describing the phenomenon in 80% of cases [11]. However, several differences in study design such as population size (n = 10), time between onset of symptoms and MRI (6 to 120 days), and magnetic field strength (1.0 Tesla) make a direct comparison difficult. Furthermore, the main finding of a reduced CBV in patients with CCD could not be reproduced in the series published by Lin et al. [12] nor in the present series. The detection rate of CCD is also lower in comparison to several PET studies [7], [27] which may be at least partially explained by the lower sensitivity of perfusion MRI techniques compared to PET to depict subtle hemodynamic alterations [28].

The observation of CCD in acute isolated thalamic infarctions underscores that the phenomenon may be present even in small ischemic lesions and not exclusively in larger territorial infarctions. While CCD has been observed predominantly in patients with large infarctions in the middle cerebral artery territory [7], [23], the relation of hemodynamic alterations in CCD and infarction size is still a matter of debate [29]. Notably, in the study of Lin et al. only two of 47 patients with CCD had small thalamic infarctions [12]. However, the authors did not report the frequency of isolated thalamic infarctions in their study population and consequently, this finding might be the result of a selection bias. In the study of Yamada et al. all patients had territorial infarctions or intracerebral hemorrhage in the basal ganglia [11]. Although all patients in the present series had small ischemic lesions in comparison to territorial infarctions, we could demonstrate a significantly larger ischemic lesion size in patients with CCD compared to those with unremarkable PWI, raising the question whether a certain lesion size might be necessary for the occurrence of CCD in thalamic infarction. Nevertheless, there was no correlation of lesion size and degree of perfusion alterations as demonstrated by mean signal ratios of TTP, MTT, CBF, and CBV.

On the other hand, this finding demonstrates that in particular thalamic lesions may be prone to CCD since the thalamus is an important relay for afferent connections from cerebellar nuclei to the cerebral cortex [22], [30], [31]. Interestingly, only patients with infarction in the territory of the tuberothalamic, paramedian as well as inferolateral arteries demonstrated CCD on PWI in this series. On conventional MRI it is not possible to identify the involvement of specific thalamic nuclei in acute thalamic infarction. Nevertheless, the infarction pattern allows to draw conclusions regarding the affected vascular territory and thalamic nuclei [32]. The only thalamic nucleus supplied by all three arteries as mentioned above is the ventrolateral nucleus which is an important relay in the dentatorubrothalamic pathway [31]. Thus, involvement of the ventrolateral nucleus in thalamic stroke leading to disruption of the dentatorubrothalamic pathway and consecutive retrograde inactivation of the contralateral cerebellar hemisphere could explain the presence of CCD in these cases. This hypothesis may be supported by single case reports on CCD in patients with circumscribed thalamic hemorrhage [8], [10], [33]. Furthermore, the clinical presentation of thalamic infarction in the territory of the tuberothalamic, paramedian and inferolateral arteries frequently comprises hemiataxia of varying severity among others symptoms [16], [34], [35] while hemiataxia in thalamic infarction in the posterior choroidal arteries territory is a rare observation [16], [17]. However, the latter is a very uncommon subtype of isolated thalamic infarction [17] and is represented by one single case only in the present study. Consequently, we cannot exclude the presence of CCD in thalamic infarction in the posterior choroidal arteries territory in general.

With regard to the clinical presentation, only dysarthria was observed more frequently in patients with CCD. However, although cerebellar lesions may cause dysarthria, this is not a characteristic finding. In particular thalamic infarctions may be the cause of dysarthria and consequently, we cannot prove a relation of this symptom to CCD.

The present study has some limitations. First, this is a retrospective case series of moderate size including 9 cases with CCD. Second, only a subset of patients with CCD underwent follow-up MRI and of these only one patient had follow-up PWI. Thus, we cannot draw any conclusions about the evolution of perfusion abnormalities in CCD with time. Third, compared to PET perfusion MRI techniques have a lower sensitivity to depict subtle hemodynamic alterations and as a consequence, some cases of CCD might have been missed. Another limitation of perfusion MRI is the restricted spatial resolution and susceptibility to bone artifacts especially in the posterior fossa.

In conclusion, multimodal MRI including DWI and PWI detects CCD in acute isolated thalamic in about 20% of patients. While previous studies demonstrated mainly a relation of CCD to large supratentorial infarction or hemorrhage, the results of our study suggest that CCD is a frequent finding in acute isolated thalamic infarctions. Furthermore, we could demonstrate that ischemic lesion size may be a relevant factor in the pathophysiology of CCD.
